# Calycosin plays a protective role in diabetic kidney disease through the regulation of ferroptosis

**DOI:** 10.1080/13880209.2022.2067572

**Published:** 2022-05-19

**Authors:** Di Huang, Peicheng Shen, Chen Wang, Jiandong Gao, Chaoyang Ye, Feng Wu

**Affiliations:** a TCM Institute of Kidney Disease, Shanghai University of Traditional Chinese Medicine, Shanghai, China; b Department of Nephrology, Shuguang Hospital Affiliated to Shanghai University of Traditional Chinese Medicine, Shanghai, China

**Keywords:** High glucose, lipid reactive oxygen species, glutathione peroxidase 4

## Abstract

**Context:**

Diabetic kidney disease (DKD) is a devastating complication of diabetes. Renal functional deterioration caused by tubular injury is the primary change associated with this disease. Calycosin shows protective roles in various diseases.

**Objectives:**

This study explored the function and underlying mechanism of calycosin in DKD.

**Materials and methods:**

HK-2 cells were treated with 25 mM high glucose (HG) to establish a renal tubule injury cell model. Then, the viability of cells treated with 0, 5, 10, 20, 40 and 80 μM of calycosin was measured using Cell Counting Kit-8. For the *in vivo* model, db/db mice were treated with 10 and 20 mg/kg/day of calycosin; db/m mice served as controls. The histomorphology was analyzed via haematoxylin and eosin staining.

**Results:**

HG-induced decreased expression of glutathione (491.57 ± 33.56 to 122.6 ± 9.78 μmol/mL) and glutathione peroxidase 4 (inhibition rate 92.3%) and increased expression of lactate dehydrogenase (3.85 ± 0.89 to 16.84 ± 2.18 U/mL), malondialdehyde (3.72 ± 0.66 to 18.2 ± 1.58 nmol/mL), lipid ROS (4.31-fold increase) and NCOA4 (7.69-fold increase). The effects induced by HG could be blocked by calycosin. Moreover, calycosin alleviated the HG-induced decrease of cell viability and the increase of lipid ROS, but erastin could block the effects caused by calycosin. The *in vivo* model showed that calycosin alleviated the renal injury caused by diabetes.

**Discussion and conclusion:**

Calycosin has a protective effect on diabetic kidney disease; ferroptosis may be involved in this process.

## Introduction

Diabetic kidney disease (DKD) is a renal microvascular disease that is caused by and known as one of the most devastating complications of diabetes (Shi and Hu 2014). It is also the leading cause of end-stage renal disease. Renal functional deterioration caused by tubular injury is considered the primary change associated with the disease (Watanabe et al. 2016). The complications of diabetes, including DKD, threaten people’s health worldwide. The current treatments for diabetes such as the control of blood glucose, blood lipids and blood pressure are not satisfactory. Therefore, more effective targets for preventing or delaying the progress of DKD must be explored. Many mechanisms are associated with hyperglycemia-induced tubular injury, including the increase in extracellular matrix expression, Wnt/β-catenin activity and generation of advanced glycation end products (Kanwar et al. 2011; Hu et al. 2015). Additionally, ferroptosis is demonstrated to damage renal tubules in diabetic models through the HIF-1α/HO-1 pathway (Feng et al. 2021). Exploring the pathogenesis of DKD has an important medical value for its treatment.

As a form of regulated cell death, ferroptosis together with apoptosis, pyroptosis and necrosis are widely studied. However, ferroptosis is genetically, biochemically and morphologically distinct from other forms of cell death (Yagoda et al. 2007). It is driven by the accumulation of iron-dependent lipid reactive oxygen species (ROS) (Lu et al. 2017). The loss of glutathione peroxidase 4 (GPX4) activity and glutathione (GSH) depletion are the main causes of ferroptosis. Erastin, a ferroptosis inducer, is demonstrated to induce ferroptosis through the inhibition of GPX (Dolma et al. 2003). Accumulating evidence has shown that ferroptosis plays a crucial role in cancer cells by enhancing the sensitivity of chemotherapeutic drugs and inhibiting tumour growth (Hangauer et al. 2017; Friedmann Angeli et al. 2019). Currently, researchers pay more attention to ferroptosis-inducing drugs, which are showing promising potential in the therapy of cancers and other human diseases.

Calycosin (C16H12O5) is an isoflavone extracted from Astragali Radix (AR, Huangqi in Chinese), the dried root of *Astragalus membranaceus* (Fisch.) Bge. or A. membranaceus (Fisch.) Bge. var. *mongholicus* (Bge.) Hsiao, and is a widely used traditional medicinal plant in Asia. AR possesses a long clinical history in the treatment of various diseases, including diabetic nephropathy (Gao et al. 2012; Ren et al. 2016). As the predominant component of AR, calycosin exhibits immunomodulatory, anti-inflammatory, antiviral and antioxidant properties (Nie et al. 2016). This study determined the protective role of calycosin in a high glucose (HG)-induced renal tubule injury model.

## Materials and methods

### Cells and cell culture

The human proximal tubular epithelial cell line HK-2 was purchased from ATCC (Manassas, VA, USA) and cultured in DMEM/F12 medium according to the manufacturer’s recommendations.

For the diabetic cell model, HK-2 cells were treated with 25 mM d-glucose (Sigma, St. Louis, MO, USA) for 24 h and 25 mM mannitol (Sigma) was used as control. Then, the cells were treated with different concentrations (0, 5, 10, 20, 40 and 80 μM) of calycosin (MedChemExpress, Monmouth Junction, NJ, USA).

### Measurement of lactate dehydrogenase (LDH), malondialdehyde (MDA), GSH, blood urea nitrogen (BUN) and creatinine (Cr)

The levels of LDH, MDA, GSH, BUN and Cr were measured using the kits from Jiancheng Biotech (Nanjing, China) according to the manufacturer’s protocols.

### Measurement of lipid ROS

The lipid ROS levels were measured using the C11-BODIPY kit (D3861, Thermo Fisher Scientific Inc., Grand Island, NY, USA). The treated cells were cultured with 10 mM C11-BODIPY for 1 h. Then, the cells were harvested and washed with PBS containing 1% bovine serum albumin. The lipid ROS levels were determined using a flow cytometer (BD Biosciences, San Jose, CA, USA).

### Cell viability

The cell viability was analyzed using the Cell Counting Kit-8 (CCK-8, Beyotime, Shanghai, China) assay. A total of 1 × 10^5^ cells were seeded into each well of a 96-well plate. After treatments, 10 μL of CCK-8 was added to each well and incubated for 1 h. The cell viability was analyzed by measuring the absorbance at 450 nm using a microplate reader (Bio-Rad Laboratories, Inc., Hercules, CA, USA).

### Real-time PCR

The relative expression levels of GPX4 and NCOA4 were detected using a real-time PCR assay. An RNeasy Mini Kit (Invitrogen, Carlsbad, CA, USA) and an M-MLV Reverse Transcriptase kit (Promega, Madison, WI, USA) were used for RNA extraction and cDNA synthesis, respectively. Then, SYBR^®^ Green (Thermo Fisher Scientific) was applied for PCR assay on an ABI7500 system (Applied Biosystems, Foster City, CA, USA). β-actin served as the internal control, and the 2^−ΔΔCT^ method was used to calculate the relative gene expression levels.

**Table:** 

GPX4	Primer F	5′-GTTACTCCCTGGCTCCTG-3′
	Primer R	5′-CTCCCAGTGAGGCAAGAC-3′
NCOA4	Primer F	5′-TGATCTCCAACCTTTTCC-3′
	Primer R	5′-CTTACATACCCAGCACCG-3′
β-actin	Primer F	5′-GATGACCCAGATCATGTTTGAG-3′
	Primer R	5′-TAATGTCACGCACGATTTCC-3′

### Western blot

The total cell lysis was obtained by adding radioimmunoprecipitation assay (RIPA) lysis buffer (Bio-Rad Laboratories, Inc.) to cells. For the pancreatic tissues, they were washed and homogenized in RIPA buffer. After measuring the protein concentrations by using a bicinchoninic acid Protein Assay Kit (Bio-Rad Laboratories, Inc.), the proteins were separated on 10% sodium dodecyl sulfate-polyacrylamide gel electrophoresis, followed by blotting on polyvinylidene difluoride membrane (Millipore Corp., Bedford, MA, USA). Then, the membranes were blocked with 5% BSA, incubated with the primary and secondary antibodies, and developed using an ECL system (Bio-Rad Laboratories, Inc.). Details of the primary antibodies are as follows:

Anti GPX4, Abcam, Ab125066;

Anti NCOA4, Abcam, Ab86707;

Anti β-actin, Abcam, Ab8226.

### Lentivirus

Knockdown lentivirus against GPX4 (shGPX4-1, GGAUGAAGAUCCAACCC; shGPX4-2, GGAGUAACGAAGAGAUC; shGPX4-3, GAGGCAAGACCGAAGUA; shNC, UUCUCCGAACGUGUCACGUTT) was purchased from GeneChem Company (Shanghai, China).

### Animal experimental design

Eighteen diabetic male db/db mice (8 weeks old) and six db/m mice (8 weeks old) were purchased from Shanghai SLAC Laboratory Animal Co., Ltd. (Shanghai, China). The db/db mice were randomly divided into a db/db group, a db/db plus 10 mg/kg/day calycosin group, and a db/db plus 20 mg/kg/day calycosin group. The db/m mice served as controls. Calycosin or saline was intraperitoneally injected into the mice for 4 weeks. At the end of the treatment, the mice were sacrificed. All animal experiments were approved by the Animal Ethics Committee of Shanghai University of Traditional Chinese Medicine (PZSHUTCM18102604).

### Haematoxylin and eosin (HE) staining assay

The kidney tissues were taken from the mice of different groups and immersed in 4% paraformaldehyde for 24 h. Then, they were rinsed, dehydrated with gradient ethanol, and cut into sections. Haematoxylin was added to the sections and incubated for 10 min, followed by eosin for 1 min. Images were taken using an optical microscope (Nikon, DS-Ri2).

### Masson’s trichrome staining assay

Masson’s Trichrome staining was performed using a Trichrome Stain (Masson) Kit (Sigma) according to the user’s instructions. Images were taken using an optical microscope (Nikon, DS-Ri2).

### Statistical analysis

All data were shown as the mean ± SEM. The statistical analysis was conducted using a one-way analysis of variance and Student’s *t*-test, followed by the Newman–Keuls multiple comparison test using GraphPad Prism 5.0 (GraphPad Software, San Diego, CA, USA).

## Results

### Calycosin plays a protective role in HG-induced renal tubule injury

**Figure 1.**
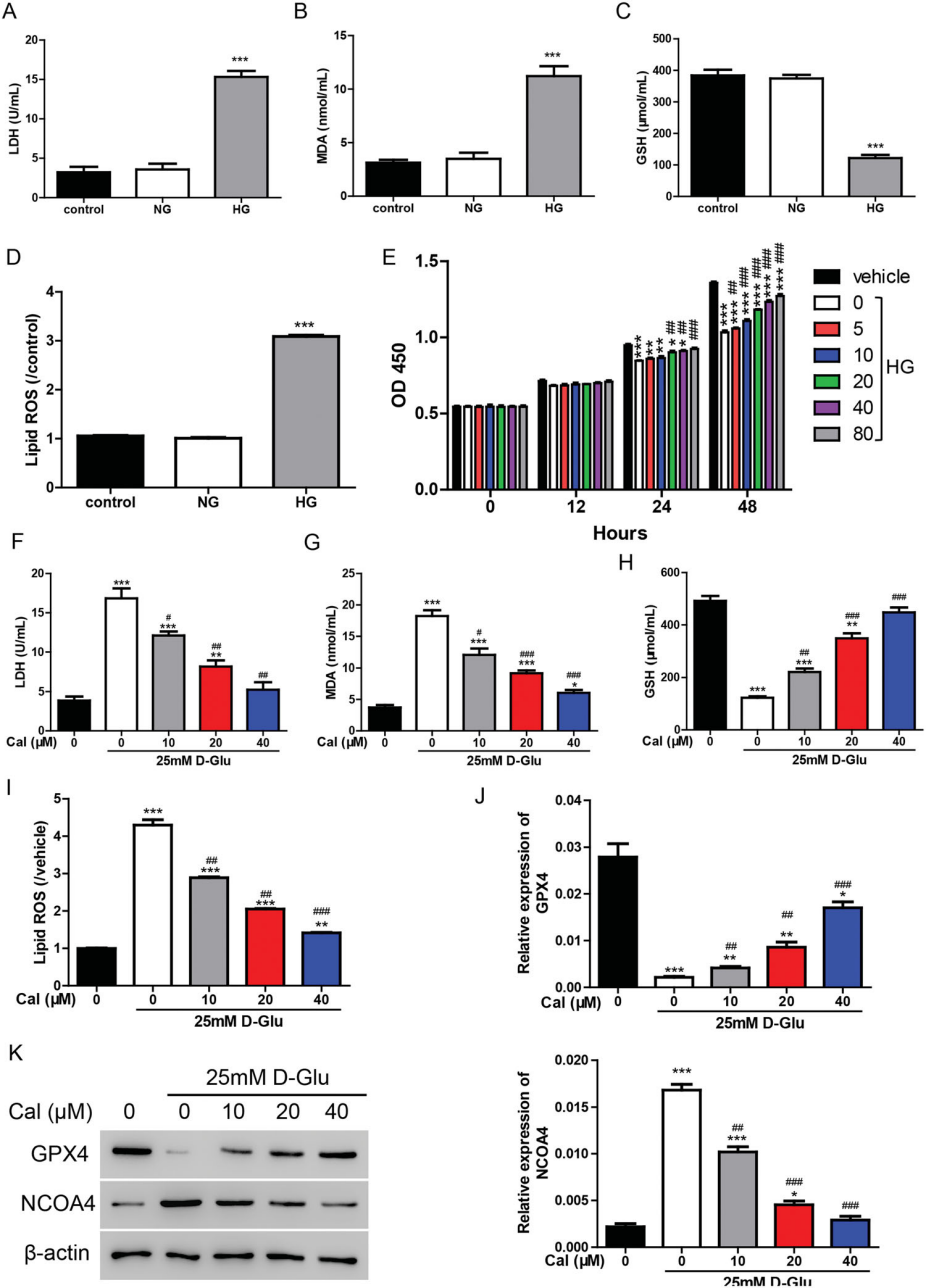
Calycosin plays a protective role in HG-induced renal tubule injury. (A–D) The levels of LDH (A), MDA (B), GSH (C) and lipid ROS (D) were measured in HK-2 cells treated with NG or HG. ***p < 0.001 vs. control. (E) The cell viability was measured in HK-2 cells treated with HG and different concentrations (0, 5, 10, 20, 40 and 80 μM) of calycosin. *p < 0.05, **p < 0.01, ***p < 0.001 vs. vehicle; ##*p* < 0.01, ###*p* < 0.001 vs. HG. (F–I) The levels of LDH (F), MDA (G), GSH (H) and lipid ROS (I) were measured in HK-2 cells treated with HG and different concentrations (0, 10, 20 and 40 μM) of calycosin. *p < 0.05, **p < 0.01, ***p < 0.001 vs. vehicle; #*p* < 0.05, ##*p* < 0.01, ###*p* < 0.001 vs. HG. (J, K) mRNA (J) and protein (K) levels of GPX4 and NCOA4.

To explore the function of calycosin in HG-induced renal tubule injury, HK-2 cells were cultured with 25 mM glucose to establish an *in vitro* experimental model. The LDH results showed that the LDH levels were much higher in the HG group (15.32 ± 1.31 U/mL) than in the normal glucose (NG) group (3.56 ± 1.29 U/mL) (Figure 1(A)), suggesting that HG treatment-induced cell injury in HK-2 cells. Ferroptosis has been demonstrated to participate in renal tubular cell death in diabetic nephropathy (Wang et al. 2020). Herein, the levels of MDA, GSH and lipid ROS upon HG treatment were measured. As shown in Figure 1(B–D), HG treatment increased the expression of MDA (from 3.49 ± 0.99 to 11.22 ± 1.59 nmol/mL) and lipid ROS (3.06-fold increase) and decreased the expression of GSH (from 374.26 ± 20.46 to 121.94 ± 16.85 μmol/mL), indicating that ferroptosis was involved in the HG-induced cell injury.

We then examined the effect of calycosin on HG-induced renal tubule injury by measuring cell viability. Different concentrations (0, 5, 10, 20, 40 and 80 μM) of calycosin were applied to HG-cultured HK-2 cells. As shown in Figure 1(E), incubation with HG significantly inhibited the cell viability (inhibition rate 23.91%), whereas calycosin increased the cell viability in a dose-dependent manner. In the following studies, 10, 20 and 40 μM of calycosin were used to treat cells. The LDH results exhibited that calycosin blocked the cell injury induced by HG (Figure 1(F)). Moreover, the increase of MDA and lipid ROS and the decrease of GSH content were alleviated by calycosin (Figure 1(G–I)). Additionally, we detected the mRNA and protein levels of GPX4 and NCOA4, two important markers of ferroptosis. We found that GPX4 (inhibition rate 92.3%) was suppressed, and NCOA4 (7.69-fold increase) was promoted in HG-cultured HK-2 cells, but calycosin could block these effects (Figure 1(J,K)). Taken together, ferroptosis may contribute to the protection of calycosin in HG-induced cell injury.

### Calycosin elicits its effects through the inhibition of ferroptosis

**Figure 2.**
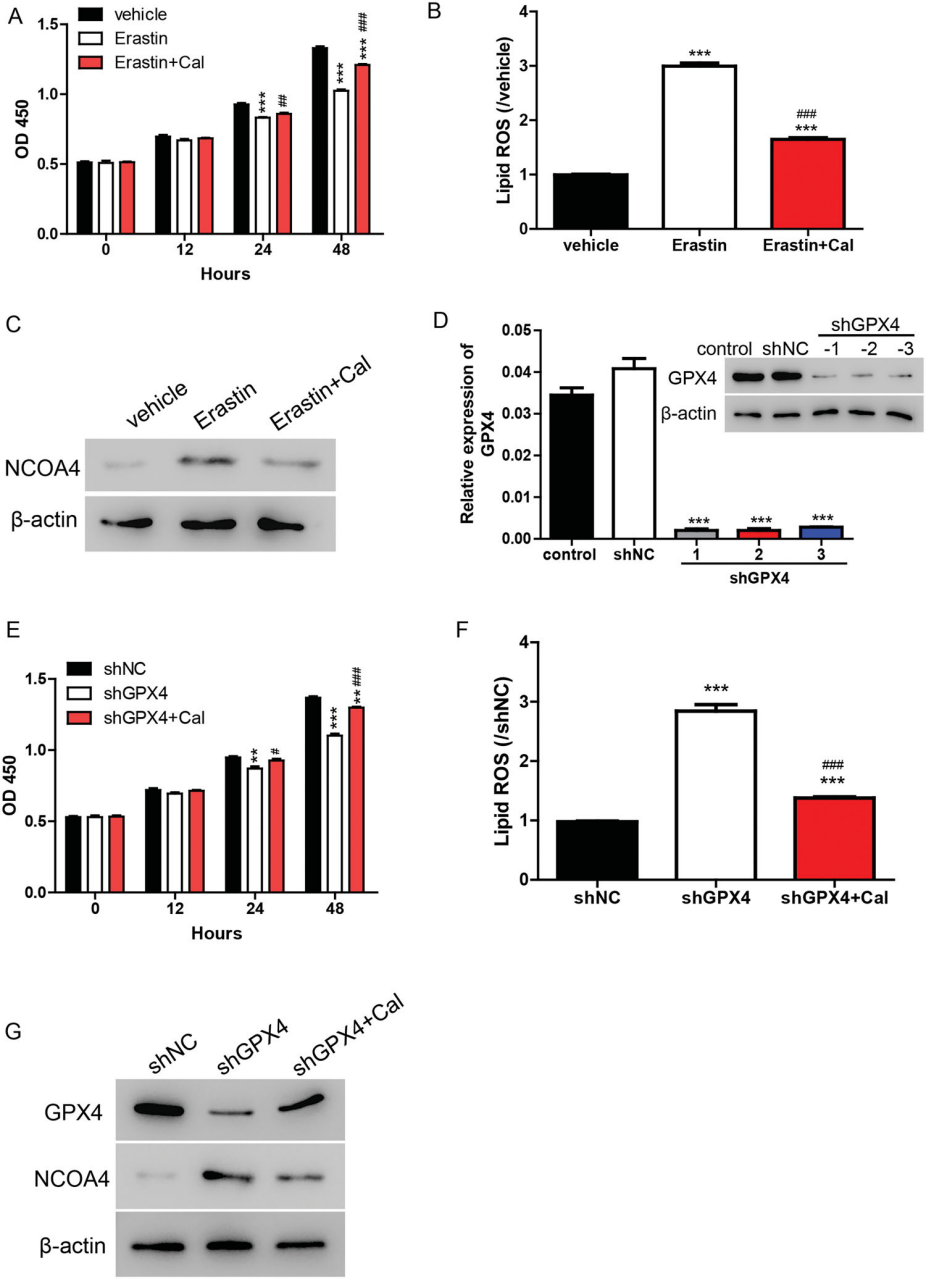
Calycosin elicits its effects through the inhibition of ferroptosis. (A–C) HK-2 cells were treated with erastin (35 μM) and/or calycosin (40 μM). Then, the cell viability (A), lipid ROS levels (B) and NOCA4 protein levels (C) were detected. ***p < 0.001 vs. vehicle; ##*p* < 0.01, ###*p* < 0.001 vs. erastin. (D) Three knockdown lentiviruses against GPX4 (shGPX4-1, shGPX4-2 and shGPX4-3) were transfected to HK-2 cells, and GPX4 expression level was measured by real-time PCR and Western blot. ***p < 0.001 vs. shNC. (E–G) HK-2 cells were transfected with shGPX4 lentivirus and/or calycosin (40 μM). Then, the cell viability (E), lipid ROS levels (F), and NOCA4 protein levels (G) were detected. **p < 0.01, ***p < 0.001 vs. shNC; #*p* < 0.05, ###*p* < 0.001 vs. shGPX4.

To verify whether ferroptosis is involved in the function of calycosin, erastin (35 μM), an inducer of ferroptosis and/or calycosin (40 μM) was used to treat cells. The cell viability results showed that erastin significantly inhibited the cell viability, whereas calycosin alleviated this effect (Figure 2(A)). Furthermore, we measured the lipid ROS levels and found that the increase of ROS induced by erastin was blocked by calycosin (Figure 2(B)). Additionally, the protein levels of NCOA4 were increased by erastin and decreased by calycosin (Figure 2(C)).

As GPX4 is the key member of the ferroptosis pathway, knockdown lentivirus targeting GPX4 was applied to further confirm whether ferroptosis is involved in the function of calycosin. According to Figure 2(D), shGPX4 lentiviruses markedly depressed the mRNA and protein levels of GPX4. Moreover, the knockdown of GPX4 significantly inhibited the cell viability and induced lipid ROS production and NCOA4 expression in HK-2 cells. However, these effects were alleviated by calycosin (Figure 2(E–G)), indicating that calycosin elicited its effects through the inhibition of ferroptosis.

### Calycosin increases the cell viability in HG-induced renal tubule injury via inhibiting ferroptosis

**Figure 3.**
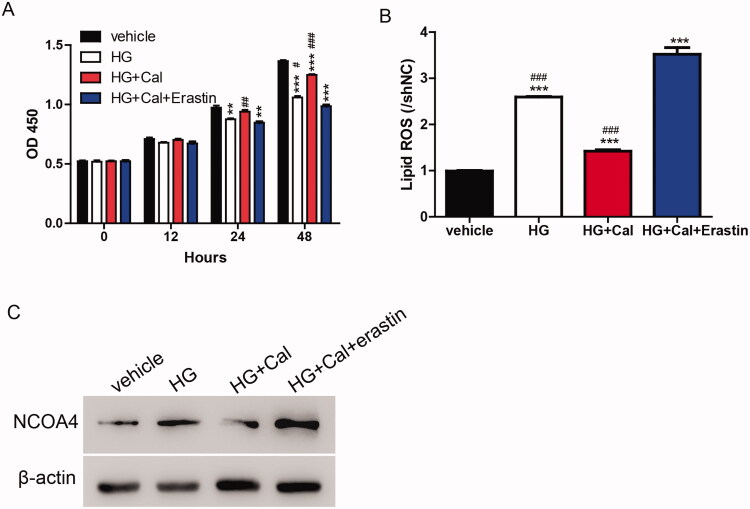
Calycosin increases cell viability in HG-induced renal tubule injury via inhibiting ferroptosis. (A–C) HK-2 cells were treated with erastin (35 μM) and/or calycosin (40 μM) upon pre-treatment with HG. Then, the cell viability (A), lipid ROS levels (B) and NOCA4 protein levels (C) were detected. **p < 0.01, ***p < 0.001 vs. vehicle; #*p* < 0.05, ##*p* < 0.01 and ###*p* < 0.001 vs. HG + Cal + Erastin.

Based on the results shown in Figure 2, calycosin elicited its function through the regulation of ferroptosis. We determined whether this mechanism could be transferred to an HG-induced renal tubule injury cell model. As shown in Figure 3(A), the HG-induced inhibition of cell viability was partially blocked by calycosin compared with the vehicle. Compared with HG, the increase in cell viability caused by calycosin was reversed by erastin. Similarly, lipid ROS and NCOA4 levels were promoted upon HG treatment compared with the vehicle. HK-2 cells incubated with HG showed lower levels of lipid ROS and NCOA4 than cells treated with HG, calycosin, and erastin but still higher than cells treated with HG and calycosin (Figure 3(B,C)). These results suggested that ferroptosis contributed to the function of calycosin in HG-induced renal tubule injury.

### Calycosin mitigates the kidney injury in db/db mice

**Figure 4.**
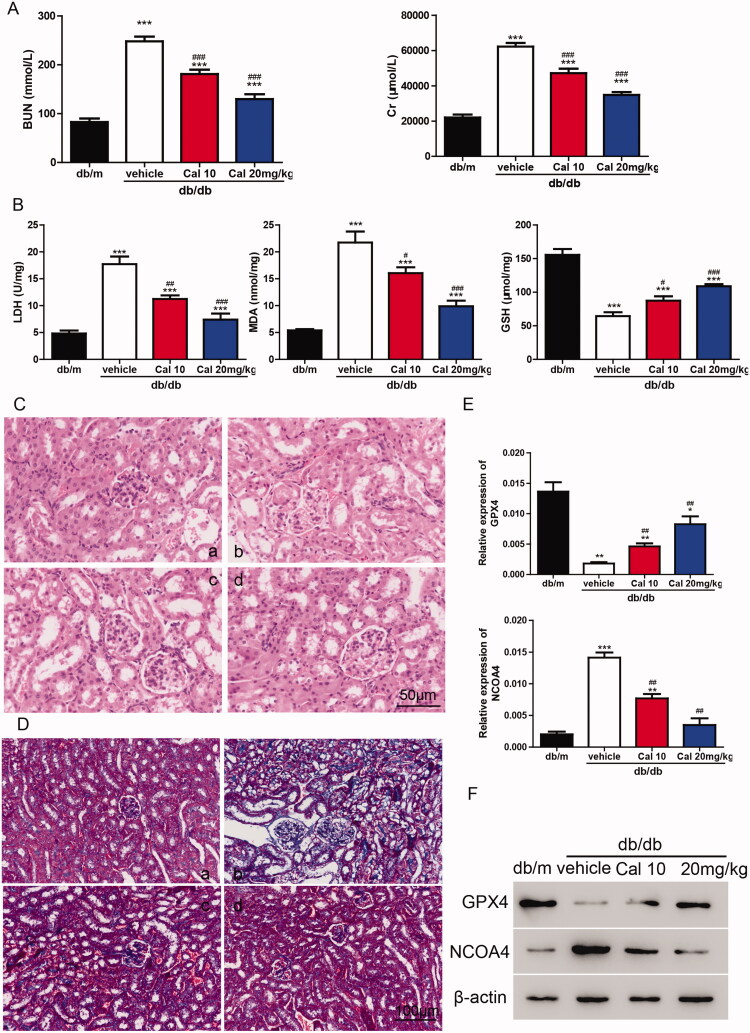
Calycosin plays a protective role in db/db mouse through the inhibition of ferroptosis. (A) Levels of BUN and Cr. (B) Levels of LDH, MDA and GSH. ***p < 0.001 vs. db/m; #*p* < 0.05, ##*p* < 0.01, and ###*p* < 0.001 vs. db/db vehicle. (C, D) HE (C) and Masson’s Trichrome (D) staining of kidney sections. (a) db/m mice; (b) db/db mice; (c) db/db mice treated with 10 mg/kg of calycosin; (d) db/db mice treated with 10 mg/kg of calycosin. (E, F) The mRNA (E) and protein (F) levels of GPX4 and NCOA4 were detected. *p < 0.05, **p < 0.01, ***p < 0.001 vs. db/m and ##*p* < 0.01, vs. db/db vehicle.

To verify the roles of calycosin *in vivo*, db/db mice were treated with 10 or 20 mg/kg of calycosin. As shown in Figure 4(A), db/db mice showed significantly higher levels of BUN and Cr compared with db/m mice. Interestingly, db/db mice treated with calycosin showed decreased levels of BUN and Cr compared with db/db mice without treatment. Furthermore, compared with db/m mice, db/db mice exhibited higher LDH and MDA levels and lower GSH levels. However, these effects were alleviated by calycosin (Figure 4(B)). To explore the renal morphological changes, HE staining and Masson’s Trichrome staining was performed. According to the HE staining results, tubular dilatation and loss of proximal tubule brush border were found in db/db mice. The less tubular injury was observed in the db/db mice treated with calycosin (Figure 4(C)). Moreover, collagen deposition was much more serious in db/db mice than in mice treated with calycosin (Figure 4(D)). Additionally, we detected the levels of GPX4 and NCOA4 and found that GPX4 was downregulated, whereas NCOA4 was upregulated in db/db mice. Calycosin partially reversed the changes of GPX4 and NCOA4 in db/db mice (Figure 4(E,F)), indicating that ferroptosis may be involved in this process. In summary, serious kidney injury was found in db/db mice, and calycosin could mitigate the injury.

## Discussion

Calycosin, the major bioactive chemical in the dry root extract of AR, shows antitumor (Zhang et al. 2012), neuroprotective (Guo et al. 2012) and anti-inflammatory effects in humans (Hoo et al. 2010). Although two herbal formulae containing AR have been found to rescue 85% of legs that have been condemned to amputation because of nonhealing chronic diabetic ulcers (Leung et al. 2008), the role of calycosin in DKD is still poorly understood. Investigating the mechanisms contributing to DKD will be beneficial in controlling this fatal disease.

In the present study, we successfully constructed an *in vitro* DKD cell model by treating HK-2 cells with HG to mimic the disease-induced tubular injury (Figure 1). By using this cell model, we investigated the protective role of calycosin. However, the mechanisms by which HG-induced ferroptosis induces tubular injury still need to be explored.

To maintain homeostasis and avoid the onset of neoplastic disease, organ and cell survival requires the balance of cell proliferation and cell death (Hanahan and Weinberg 2011). Apoptotic and several nonapoptotic cell death pathways, including necroptosis, pyroptosis and ferroptosis, are activated to eliminate damaged and unnecessary cell components (Galluzzi et al. 2018). In contrast to the role of apoptosis in tumour suppression, the contributions of nonapoptotic cell death pathways in tumour suppression have only been discovered recently (Jiang et al. 2015; Wang et al. 2017).

Ferroptosis is an iron-dependent form of cell death. In this process, free iron or lipoxygenase enzymes containing iron oxidize polyunsaturated fatty acids (PUFAs), resulting in the generation of lipid ROS. Iron–transferrin complexes are responsible for importing iron into cells. The downregulation of iron–transferrin complexes inhibits ferroptosis (Gao et al. 2015; 2016). Iron–transferrin complexes localize to the lysosome, in which iron is liberated into the cytosol. There, the majority of iron can be liberated via NCOA4-mediated ferritinophagy (Mancias et al. 2014). Upon iron-catalyzed PUFA oxidation, the (reduced) GSH-dependent lipid hydroperoxidase GPX4 could maintain homeostasis by reducing lipid hydroperoxides to lipid alcohols in membrane environments (Ursini et al. 1985). In the present study, we observed that NCOA4 was promoted, whereas GPX4 was inhibited by HG treatment in HK-2 cells (Figure 1), indicating the increased release of free iron in cells and increase of lipid ROS, both of which finally induce ferroptosis in the HK-2 cell model. However, the mechanisms by which HG-induced ferroptosis induces ferroptosis are still not clear.

Erastin induces ferroptosis, which is distinct from other forms of nonapoptotic cell death such as apoptosis and classic necrosis. To clarify the protective effects of calycosin, we induced ferroptosis using erastin and observed that calycosin relieved the induction of ferroptosis by reducing lipid ROS, LDH and NCOA4 levels (Figure 3). Erastin induces ferroptosis by inhibiting GSH and GPX4, which is consistent with our findings (Figure 3). However, we also observed that NCOA4 changed during ferroptosis induction. These data raise the possibility that iron transportation should also contribute to ferroptosis in HG-induced ferroptosis in the HK-2 cell model. However, the findings in the DKD mouse model need to be verified. Moreover, the protective roles of calycosin need to be evaluated in a mouse model.

## Conclusions

Our results demonstrated that calycosin rescued HG-induced ferroptosis in HK-2 cells. Mechanistically, calycosin inhibited ferroptosis induced by HG via reducing lipid ROS and free iron import in HK-2 cells.
